# Knowledge Maps and Emerging Trends in Cell-Cultured Meat since the 21st Century Research: Based on Different National Perspectives of Spatial-Temporal Analysis

**DOI:** 10.3390/foods13132070

**Published:** 2024-06-29

**Authors:** Huiqi Song, Pengwei Chen, Yiwen Sun, Jiping Sheng, Lin Zhou

**Affiliations:** 1 School of Agricultural Economics and Rural Development, Renmin University of China, Beijing 100872, China; songhuiqi2020@163.com; 2 Academy of Animal Husbandry and Veterinary Sciences, Qinghai University, Xining 810016, China; chenpengweixuexi@163.com; 3 Institute of Food and Nutrition Development, Ministry of Agriculture & Rural Affairs, Beijing 100081, China; yver_sun@163.com

**Keywords:** cell-cultured meat, Citespace, Vosviewer, bibliometrics, alternative protein, visual analysis, nomenclature

## Abstract

Cell-cultured meat holds significant environmental value as an alternative protein source. Throughout the 21st century, cell-cultured meat has progressively penetrated commercial markets. However, a systematic review encompassing the entire field needs improvement. Employing Citespace, Vosviewer, and R-Bibliometrix software, a bibliometric analysis was used to present the research progress and general development trends of 484 articles on cell-cultured meat from 2000 to 2022 based on countries, authors, institutions, and keywords. This analysis provides ideas for the future development of cell-cultured meat in different countries or regions worldwide. Research on cell-cultured meat from 2000 to 2022 has undergone two phases: fluctuating growth (2000–2013) and rapid growth (2013–2022). Noteworthy contributions to cell-cultured meat studies emerge from author groups in the United States of America, the United Kingdom, and China, with influential institutions like the University of Bath significantly impacting pertinent research. Furthermore, over the past two decades, research has leaned towards exploring topics such as “biomaterials”, “cultured”, “land use”, “public opinion”, “animal welfare”, and “food safety”. Furthermore, this study reveals differences in nomenclature between regions and institutions. “Cultured meat” is more popular in some countries than in other forms. Institutions in Asia use “cultured meat” more frequently; however, institutions in the Americas adopt “cultivated meat” and rarely adopt “in vitro meat”, and institutions in the European region have no particularly prominent tendency towards a specific nomenclature. Future research should emphasize aligning the labeling of cell-cultured meat with effective management strategies and referencing regulatory policies across various countries. For the first time, we use three different bibliometric methods to analyze temporal and spatial variation in research on cellular meat. The results of this study have a multiplier effect. We provide a theoretical basis and a practical reference for the identification of alternatives in the dual context of “food crisis and food security” and “climate crisis”. At the same time, we also provide a reference for the sustainable development of the food system.

## 1. Introduction

According to the Food and Agriculture Organization of the United Nations (FAO), the global population will reach 9.5 billion by 2050. Over 70 percent of alternative proteins will be necessary to address this growth and fulfill food and nutritional demands [1,2]. Among the many novel food needs, meat has grown substantially in total and per capita consumption worldwide in recent years [3,4]. Nevertheless, evidence highlights the unsustainability of the current meat production system. So, we should explore an alternative approach to meat production that mitigates environmental strain without compromising sustainable consumption [5,6,7]. Cell-cultured meat grown from animal cells has emerged as a proposed solution to these challenges [8]. Developers perceive it as having potential advantages for the environment, animal welfare, and human health [9,10]. While the development of cell-cultured meat has entered a marketable phase after the 21st century [11], comprehending its overall evolution remains a challenge.

Previous studies of cell-cultured meat have predominantly concentrated on technological advancements [12,13], consumer perceptions [14], and environmental impact [15,16], among other aspects. Initial research primarily explored consumer satisfaction [14], consumption patterns [17,18], opportunities [19,20], life cycle assessment [21], technology preferences [22], ethical considerations [23], and related themes. However, recent scholarship has shifted its focus toward animal welfare [24], regulatory policies [9], consumer aversion [25], acceptance attitudes [26,27,28], technological innovations [29,30], and the ecological value of cultured meat [31]. A few scholars explored the nomenclature preference for cell-cultured meat in individual countries [32,33]. While the present study conducts a macro-analysis of cell-cultured meat’s development, it is necessary to examine the overall evolution of the industry comprehensively.

Scholars have explored cell-cultured meat in depth from the perspectives of external impacts, inherent product development technologies and characteristics, and comprehensive perspectives. First, in terms of external impacts, some scholars have explored the possible social aspects of cell-cultured meat. For example, Bryant et al. (2020) viewed the idea of cultured meat as a technology with multiple social considerations and far-reaching social implications [34]. In addition, some scholars are concerned about the environmental issues that may arise from cell-cultured meat, including land use. For example, Alexander et al. (2017) reviewed alternatives to traditional animal products. They included cultured meat, imitation meat, and insects [35]; Chriki and Hocquette (2020) opened a discussion on technical issues surrounding cellular meat and meat diversity [36]. In addition, scholars have paid special attention to the consumer acceptance of cell-cultured meat. Bryant and Barnett (2018) synthesized and analyzed the results of 14 empirical studies to conduct a systematic review of studies on consumer acceptance of cell-cultured meat [37]; Pakseresht et al. (2022) argued that the most important factors influencing consumer acceptance/rejection of cultured meat include public awareness, perception of naturalness, and perceived food-related risks [26].

Secondly, some scholars have also carried out extensive research on the intrinsic characteristics and technology of cell-cultured meat products. Among them, some scholars focus on the specific technological advances and nutritional health issues of cell-cultured meat. Ben-Arye et al. (2019) detailed the application of stem cell and tissue engineering fields [38]; Zhang et al. (2020) summarized the current technological challenges and their possible solutions based on tissue and bioreactor engineering [39]. In addition, a small number of scholars address the issue of product characterization of cell-cultured meat itself. For example, Rubio et al. (2020) detailed the advantages and challenges of plant and cellular meat alternatives in terms of production efficiency, product characterization, and impact categories [40].

Finally, many scholars have considered the nutritional, technological, social institutional, biological, regulatory, consumer, and present and anticipated future roles of cell-cultured meat that may arise from an integrated perspective. For example, Stephens et al. (2018) focused on cultured meat and its technological, socio-political, and regulatory challenges and opportunities [11]; Post et al. (2020) focused on the biological, technological, regulatory, and consumer acceptance challenges in the field of biotechnology development [9]; Van der Weele et al. (2019) applied the Reflective Integrated Comparison Heuristic (RICH) to comparatively assess five meat substitutes [41]; Santo et al. (2020) explored the combined public health impacts associated with the production and consumption of plant-based meat substitutes and cellular-based meat [42]. Additionally, some scholars have used bibliometric methods to explore the literature related to cell-cultured meat. Chriki et al. (2020) conducted a bibliometric analysis revealing the evolution of cell-cultured meat studies [43]; Fernandes et al. (2020) used Vosviewer software to categorize 109 papers on artificial meat into three dimensions [44].

Scholars have provided comprehensive overviews of cell-cultured meat’s industrial development, technological regulations, and cell sources [45,46,47]. However, there is a need for additional reviews using visual analytical methods to explore the temporal dimension and regional differences. To fill these knowledge gaps, this study aims to illuminate the temporal evolution and regional cooperation differences within the field of cell-cultured meat after the 21st century. We strive to visualize and analyze this progression to identify research trends and predict the industry’s future trajectory [48]. Accordingly, this study addresses the following inquiries: (i) trends in cell-cultured meat research over time, including the overall trend, the trend of countries, institutions, and authors, the trend of cited authors and journals, high-frequency keywords, co-present keywords, and timeline keywords change trend. (ii) Variations in research emphasis across global regions, including regional naming differences, cooperation content differences, and regulatory differences. (iii) Predictions regarding future trends in cell-cultured meat. In this study, three bibliometric methods visually analyze the literature on cell-cultured meat from 2000 to 2022.

Through the above analysis, this study presents the exciting findings on nomenclature in different regions and institutions and the differences in cell-cultured meat in regional cooperation content and regulations, which provides a reference for the theoretical system of cell-cultured meat. In addition, the individual marginal contribution of this study is that not many previous studies have used a quantitative approach to synthesize the content regarding cell-cultured meat. We differ from some scholars who only use one quantitative approach. For the first time in the field of cell-cultured meat, we use three representative quantitative analyses of bibliometrics. Meanwhile, we combine statistical and mathematical methods to map the development status of the research field [49], which helps to objectively identify the development of the industry. Furthermore, this study fills the theoretical gap of the lack of research in the field of cell -cultured meat that continues to evolve over time and space. This will help in identifying the research themes at various stages as well as the overall evolution. Finally, this study also provides a realistic reference for the sustainable development of cellular agriculture and food systems.

## 2. Data Sources and Analytical Methods

### 2.1. Data Sources

**Figure 1:**
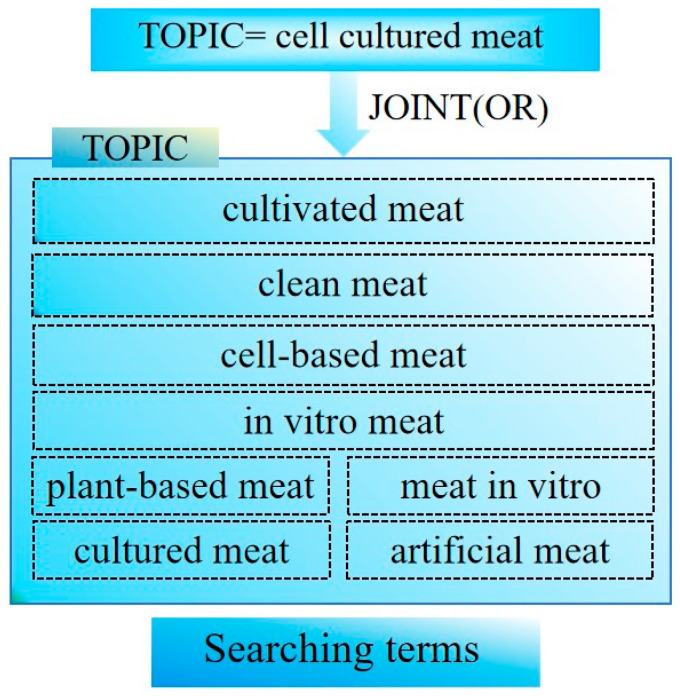
The set of search keywords.

The absence of a standardized definition leads to varied nomenclature. Post et al. (2020) highlight the controversy surrounding names like “*cultivated meat*”, “*cultured meat*”, “*cell-based meat*”, and “*clean meat*”, which differ based on the organization [9]. Post’s definition is adopted in this study, considering its relevance and citation frequency. Cell-cultured meat is the application of cell-based biotechnology to substitute traditional animal-derived products, supplement the conventional meat supply, and facilitate sustainable meat production [9,50]. Zhang (2020) is referenced for this search criteria in terms of their keywords [39,48]. Utilizing the core collection of Web of Science databases, the search encompassed terms such as “*cultured meat*”, “*in vitro meat*”, “*clean meat*”, “*artificial meat*”, “*cell-based meat*”, “*cell-cultured meat*”, “*meat in vitro*”, “*cell meat*”, “*plant-based meat*”, and “*cultivated meat*”. The search parameters were set to include papers in English that were published between 1 January 2000, and 13 September 2022. We extracted the relevant literature on 14 September 2022. The literature types were “*Article*” and “*Review Article*”. After eliminating duplicates and irrelevant articles, 484 papers cover the literature on cell-cultured meat over the last two decades. The set of search keywords is shown in Figure 1. By setting the above topic keywords, we need to address the following key research questions: (i) trends in cell-cultured meat research over time from 2000 to 2022, including the overall trend, the trend of countries, institutions, and authors, the trend of cited authors and journals, high-frequency keywords, co-present keywords, and timeline keywords change trend. (ii) Variations in research emphasis across global regions, including regional naming differences, cooperation content differences, and regulatory differences. (iii) Predictions regarding future trends in cell-cultured meat.

### 2.2. Methods of Analysis

Bibliometric analysis is a method used to quantitatively analyze literature with the help of bibliometric tools [51]. Knowledge graph analysis can reveal the knowledge structure of a research field and depict its evolution through knowledge graphs. The Citespace software, developed by Chen from Drexel University, serves as a citation network visualization tool [52]. Citespace (version 5.5 R2) is used to visualize and analyze the study of cell-cultured meat by combining knowledge graph analysis with bibliometric methods, ultimately constructing a knowledge map of the domain. Vosviewer is a free bibliometric analysis software jointly developed by Nees Jan van Eck and Ludo Waltman of Leiden University in The Netherlands to build and view bibliometric maps. It is based on the co-citation principle of literature, which can be used to map scientific maps in various knowledge fields [53]. R-Bibliometrix is a scientific bibliometric software based on R language developed by Italian scholar Dr. Massimo Aria. It is a powerful tool in creating bibliographic coupling, co-citation, co-authorship, and co-occurrence networks [54]. In this study, Citespace (version 5.5 R2), the Bibliometrix package (version 3.6.3), and Vosviewer (Version 1.6.20) are used to explore the temporal progression and spatial cooperation of cell-cultured meat.

## 3. Research Findings

### 3.1. Trends of Papers on Cell-Cultured Meat in the 21st Century

**Figure 2:**
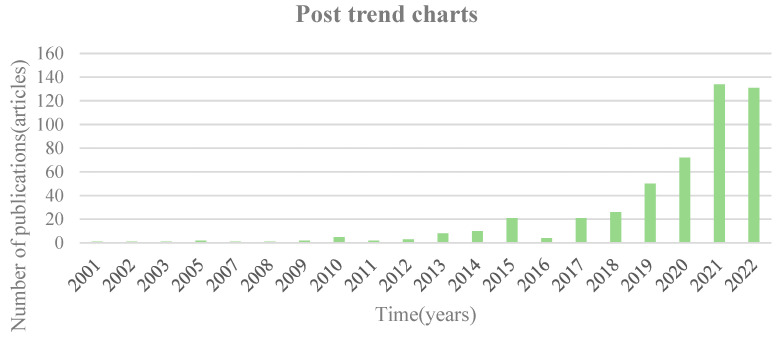
Annual distribution of cell-cultured meat publications.

The number of papers published annually served as a primary reference to assess the progression of cell-cultured meat over 22 years from 2000–2022. The literature on cell-cultured meat exhibited a fluctuating upward trend (Figure 2). Only one article related to cell-cultured meat was published in 2001, which provided a brief introduction to cell-cultured meat. By contrast, one hundred thirty-one papers were published in 2022. Based on the fluctuating trend in the literature, the study of cell-cultured meat could be categorized into two phases. The first phase spans from 2000 to 2013 and witnesses a modest number of publications, typically fewer than 20 articles. The second phase, from 2013 to 2022, marks a remarkable surge in published literature. It signifies rapid development and heightened attention towards cell-cultured meat. This phase represents a pivotal shift in the field, wherein researchers aim to mitigate the environmental impact of animal agriculture, enhance meat safety and quality, and explore alternative livestock production methods [17,20]. All in all, numerous scholars are dedicating their efforts to cell-cultured meat research. They accelerate the expansion of associated research.

### 3.2. Analysis of Issuing Countries and Institutions

**Figure 3:**
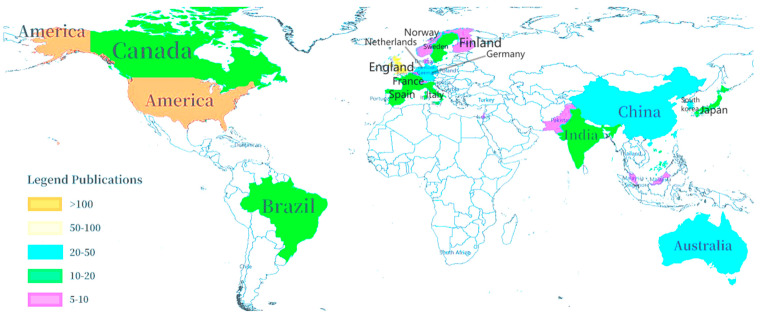
Number of published papers from countries with cell-cultured meat.

This examination delves into the inter-country and inter-institutional relationships within cell-cultured meat research. It helps us analyze the global cooperation trends and the impact of various countries on cell-cultured meat. This approach directly assesses collaborative efforts among countries and institutions [49]. The distribution of countries and the number of publications is shown in Figure 3. The results reveal authors’ contributions from 37 countries in cell-cultured meat, with a network density for collaboration at 0.2628. The top 10 countries with the most published papers on cell-cultured meat are the United States of America (the USA), the United Kingdom (the UK), China, New Zealand, Germany, Korea, Australia, Canada, France, and Italy.

The USA stands out among the nations with an impressive 123 published papers in cell-cultured meat studies. It constitutes 25.41% of the total literature and significantly surpasses the contributions of all other countries. The UK, China, and New Zealand contribute 10.95%, 8.68%, and 8.06% to the collective body of research, respectively (according to Citespace results).

**Figure 4:**
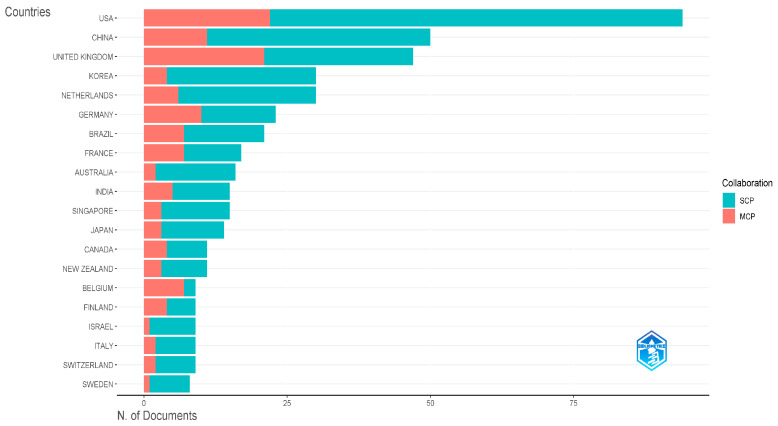
Corresponding authors’ countries of cell-cultured meat research.

According to calculations, in the network analysis, the nodes’ centrality measures the proportion of shortest paths within the network that they connect to a specific node. A higher centrality signifies greater relevance and importance of the node in the given domain. Notably, the USA and the UK exhibit centrality scores of 0.77 and 0.24. Conversely, despite their higher paper count, China and New Zealand display lower centrality scores of 0.01 and 0.05, signifying a relatively weaker level of international cooperation in cell-cultured meat research. In addition, the country cooperation profiles derived from R-language are shown in Figure 4. The single-country cooperation (SCP) and multinational-country cooperation (MCP) profiles are shown in green and orange, respectively. The results show that three countries, the United States, China, and the United Kingdom, also rank among the top three countries in the world in terms of the number of articles issued. However, overall, the number of SCP is much larger than that of MCP. Therefore, at present, most countries are still limited to intra-national cooperation, and international cooperation is yet to be further developed.

**Figure 5:**
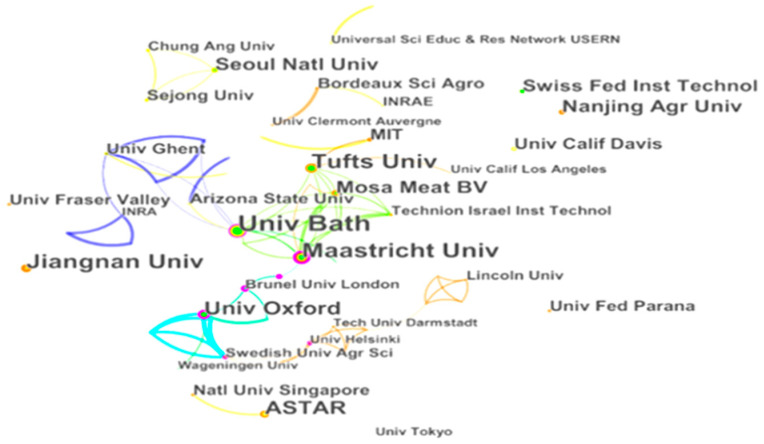
Cell-cultured meat research core academic institutions.

A concise overview of prominent international research institutions can illuminate this research domain’s distribution and collaborative networks [55]. To achieve this, we conducted a literature search on cell-cultured meat. We also used “institution” as the search term. By representing institutions as network nodes, our analysis revealed 92 key institutions actively engaged in cell-cultured meat research. The resulting graph displays these institutions as nodes connected by 127 lines, illustrating a tight research network with a density of 0.0303 (Figure 5). From 2000 to 2022, cell-cultured meat research engaged 92 prominent research institutions. In this case, the different colors indicate the collaborative networks between the different institutions. The same color indicates the existence of mutual cooperation between institutions. Institutions with a high volume of publications are indicated by a prominent red circle. Ten institutions contributed more than seven research papers, constituting 21.07% of the total publications. Bath University in the UK leads the pack regarding the number of papers published, contributing 3.30% of the total.

The other prominent institutions include Maastricht University, Tufts University, Jiangnan University, ASTAR, University of Oxford, Nanjing Agricultural University, Seoul National University, Mosa Meat B.V., and the Swiss Federal Institute of Technology. Maastricht University has the highest centrality score of 0.22, underscoring its significant influence on cell-cultured meat research.

### 3.3. Characterization of Author Groups

**Figure 6:**
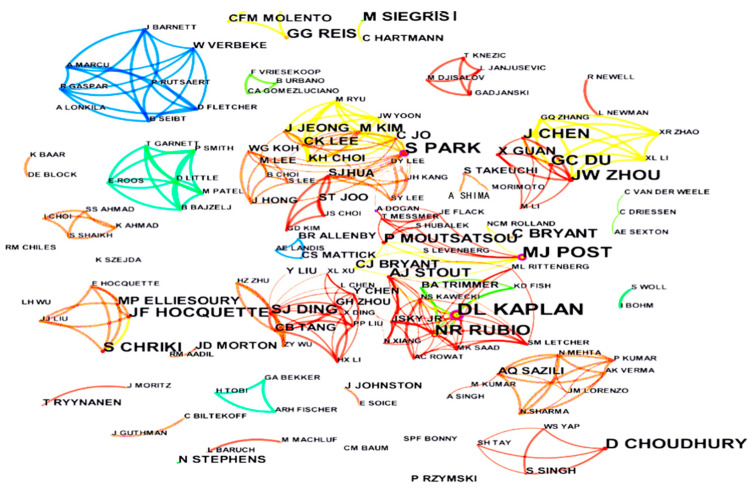
High-yielding authors of cell-cultured meat research between 2000 and 2022.

The statistics regarding authors in the field of cell-cultured meat offer valuable insights into scholars’ publications and contributions at a micro level. This data serves as a reference point for researchers in this domain, aiding in evaluating and navigating the relevant literature. By selecting the “author” option in the node-type field and configuring the time frame from 2000 to 2022, this study generates a prolific author visualization, employing clustering upon clicking “go” [55] (Figure 6).

In the visual representations, the size of each node corresponds to the number of papers published in a given year. Larger nodes signify a higher volume of publications for that specific year. The interconnecting lines between nodes represent collaborative efforts between different authors. The thickness of these lines indicates the extent of collaboration between the two authors [55]. Based on the results, several collaborative groups are identified. Specifically, Group 1 includes Post and Lee, Group 2 comprises members led by Park, and Group 3 comprises individuals associated with Kaplan and additional collaborators (Figure 6).

The collaborative networks surrounding Post and Kaplan are visually distinguished by different colors, signifying their published studies on cell-cultured meat. They also serve as a bridge between different author communities and institutions. Additionally, there is a more concentrated collaborative group with a core of leading authors who promote the discipline in the realm of cell-cultured meat. Applying the formula N_1_ = 0.749(N_max_)^1/2^ [55], we find that Kaplan stands out with 12 papers. So, we make N_max_ equal to 12 and N_1_ approximately 3. Therefore, authors who publish more than three papers are considered core contributors to cell-cultured meat. Calculations show that 49 scholars meet this criterion, constituting 40.14% of the global cell-cultured meat literature. Identifying core authors in this field typically requires them to contribute 50% of the research area’s papers. Currently, this percentage is needed to meet the relevant standards. Therefore, the research field of cell-cultured meat still needs further development.

### 3.4. Co-Cited Authors and Journals

**Figure 7:**
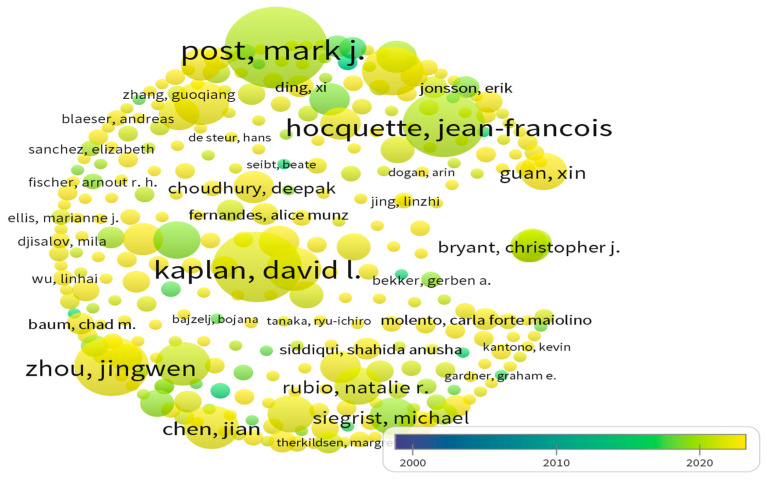
Co-cited author’s knowledge graph from 2000 to 2022.

**Figure 8:**
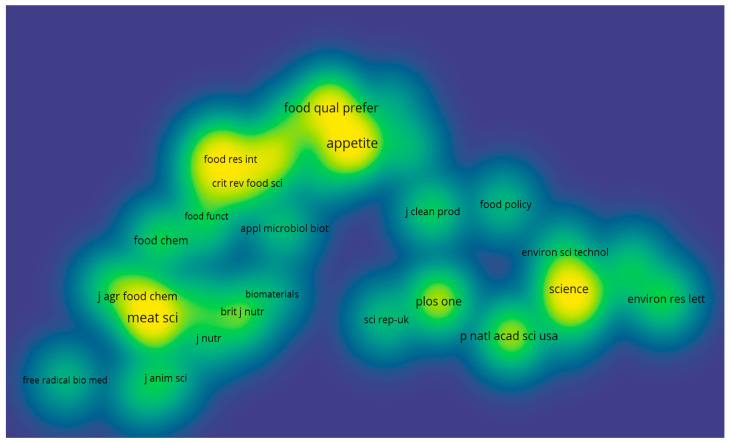
Impact maps of co-cited journals.

We utilized the “cited authors” and “cited journals” options within Citespace (version 5.5 R2) or Vosviewer software (Version 1.6.20) to comprehend the authors and publication status systematically. Subsequently, we generated network diagrams by running the cited authors and cited journals software (Table 1 and Table 2; Figure 7 and Figure 8). These diagrams encapsulate pertinent academic research within the domain of cell-cultured meat and provide comprehensive knowledge through journal co-citation analysis.

Notably, Post’s work garnered a noteworthy 452 citations, while Stephens N’s paper received 383 citations. Post [20], Kaplan [9], Zhou [39], Hocquette [36,43], Bryant [25,34], Guan [58], Chen [39], and Siegrist [27] are the most frequently cited authors. They are a core group of co-cited authors in cell-cultured meat.

According to Citespace, among the cited journals, “Trend in Food Science & Technology” stands out as the most frequently referenced, boasting 595 citations. Additionally, the distribution of co-cited journals derived from Vosviewer is shown in Figure 8. The more apparent yellow color indicates higher citation frequency, among which journals such as Appetite, Meat Science, and Science are more frequently cited.

### 3.5. High-Frequency Keyword Analysis

Keywords in a research field indicate its prevalent topics and evolving trends. To gain insights into these trends, scholars often rank keywords by their frequency of occurrence [53]. Using Citespace, our analysis selected the “keyword” option in the node type field and specified the timeframe as “2000–2022”. Meanwhile, we used Vosviewer to display high-frequency keywords.

**Figure 9:**
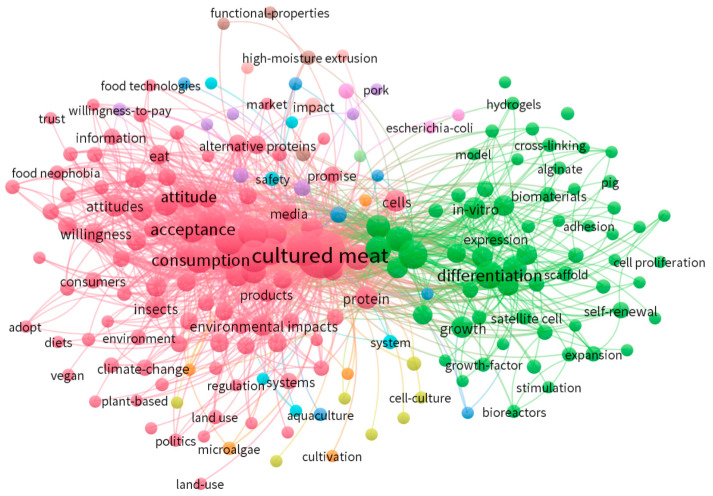
Research content knowledge mapping for high-frequency keywords between 2000 and 2022.

A total of 169 keywords from 2000 to 2022 underwent ranking based on their word frequency, reflecting the number of papers featuring each keyword. In addition, we used Vosviewer to depict the knowledge graph of high-frequency keywords, and the results are shown in Figure 9. The pink ones are related to consumption, market, and environment, and the green ones are mainly related to cell-cultured meat production and R&D technology. In addition, according to the estimation results of Citespace, the most prevalent term among these keywords is “cultured meat”, followed by “*in vitro meat*”, “*challenge*”, “*consumer acceptance*”, “*food*”, “*future*”, “*differentiation*”, “*insects*”, “*stem cells*”, “*regulatory challenge*”, “*alternative protein*”, “*technology*”, “*cellular agriculture*”, “*protein*”, “*perceived naturalness*”, “*sustainability*”, “*consumption*”, “*tissue*”, “*muscle*”, “*willingness*”, “*attitude*”, and “*acceptance*”. Specific terms exhibit substantial centrality within this array of keywords, signifying their prominence in the discourse. These pivotal keywords, including “meat”, “*food*”, “*perceived naturalness*”, “*in vitro*”, “*proliferation*”, “*greenhouse gas emissions*”, “*stem cell*”, “*regulatory challenge*”, “*cellular agriculture*”, “*consumption*”, “*health*”, “*environmental impact*”, and “*alternative*”, boast a mediated centrality of 0.07 or higher, implying their frequent mention across numerous scholarly papers.

High-frequency keywords in the realm of cell-cultured meat research span several vital areas. Initially, scholars directed their attention toward technological advancements within the field. Noteworthy terms encompass “*in vitro meat*”, “*insect*”, “*stem cell*”, “*alternative protein*”, “*technology*”, “*protein*”, “*muscle*”, “*in vitro*”, and “*proliferation*”. To illustrate, some researchers compared plant-based and cell-based meat alternatives regarding production efficiency, product characteristics, and their impact on various categories [34,42]. Meanwhile, Reiss (2021) addressed the cell source used in production in the USA, emphasizing medium composition, bioreactor expansion, and biomaterial tissue scaffolds as vital components [50].

Topics encompass rule system challenges, sustainability, greenhouse gas emissions, and environmental impacts [48]. For instance, researchers like Jasmine in Singapore focused on examining scaffolds for artificial meat. They note that the primary sources of scaffolds for cell-cultured meat are collagen and gelatin, with ongoing investigations into plant-based materials for scaffolds driven by environmental conservation and animal welfare considerations [59].

Lastly, cell-cultured meat intersects with agriculture, food, and consumption, encompassing cellular agriculture, consumer behavior, consumption patterns, and environmental impacts [60,61]. For instance, Bryant in the UK reviewed consumer acceptance of artificial meat [62]. In The Netherlands, Post examined the scientific and societal challenges of transforming artificial meat into a viable commercial option [9].

### 3.6. Keyword Clustering Analysis

**Figure 10:**
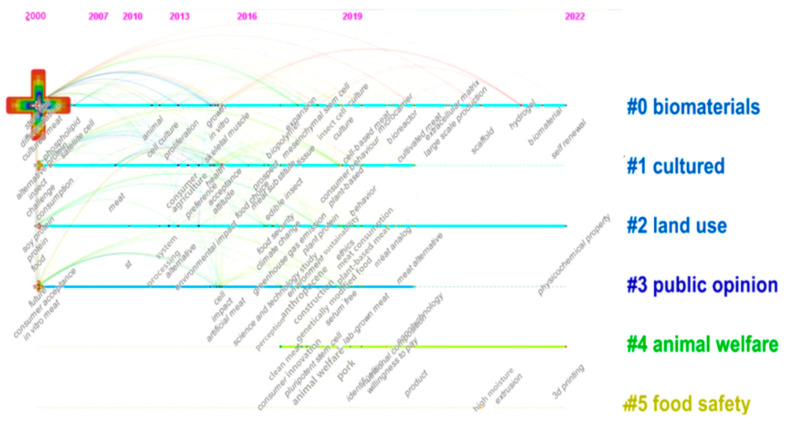
Timeline mapping of international cell-cultured meat keyword co-occurrence from 2000–2022.

Visual cluster analysis of cell-cultured meat keywords enables us to identify critical trends in the field. The timeline diagram further illustrates the relationship between these trends and the historical development of the associated literature. This paper utilizes the timeline diagram function in Citespace software, arranging keywords chronologically based on their appearance over time. The size of the annual circles along the horizontal axis indicates the frequency of citations in the literature. Additionally, cluster labels on the right side provide insights into the various topics within cell-cultured meat research [63]. Our results are shown in Figure 10.

“*#0 biomaterials*”: “*stem cell*” emerged in the year 2000, marking the inception of this exciting field. Subsequently, “*growth*” and “*in vitro culture*” appeared in 2015, while 2021 witnessed breakthroughs in “extracellular matrix utilization and large-scale production techniques”. In 2022, the authors brought fresh insights by introducing hydrogel technology and vital components in cell-cultured meat development. This progression underscores the dynamic history of technological innovation in this field. The journey of cell-cultured meat technology began with the discovery of stem cells, paving the way for in vitro cell production [38]. Scaffold biomaterials, integral to creating a supportive cell expansion and differentiation framework, have played a central role in this developmental trajectory. Nevertheless, it is essential to note that this technology is still nascent and confronts several critical challenges [29]. These include sourcing appropriate cells, addressing animal-related concerns, and optimizing bioprocessing for large-scale commercial production [11].

“*#1 cultured* “, the evolution of research into alternative protein sources, including “insects”, has notably progressed. The concept of alternative protein sources emerged in 2000, with the subsequent introduction of the term “*challenge*” in 2002. In 2014, the term “*consumer*” became a focal point of discussion. This was followed by exploring consumer food choices [64] and, most recently, examining consumer behavior concerning these alternatives [56]. The research landscape has shifted towards agriculture, particularly after the initial challenges associated with cell-cultured meat formation [19]. More recently, there has been a growing emphasis on the potential development of cell-cultured meat. This evolution in research perspectives underscores the dynamic nature of the alternative protein discourse, with a significant shift from its inception to its current focus on consumer behavior and the prospects of cell-cultured meat [33,65].

“*#2 land use*”, “*soy protein*” and “*protein*” first emerged in 2000. In 2014, concerns about “*environmental impact*” gained prominence, with subsequent years bringing attention to critical issues like “*food security*”, “*climate change*”, and “*greenhouse gas emissions*” in 2017. The term “*meat alternative*” also debuted in 2020, while exploring “*physicochemical properties*” became a focal point in 2022. In the early stages, investigations related to cell-cultured meat and land use predominantly centered on soy protein and plant-based substitutes. Over time, the focus shifted towards broader environmental concerns within the food security system, encompassing food safety [61]. Eventually, the authors’ attention expanded to macro ecosystem concerns, including climate change, greenhouse gas emissions, and other environmental factors influencing development [66,67]. More recent research has honed in on cell-cultured meat products’ physical and chemical attributes, particularly in land use, with meat consumption as a representative indicator [35].

“*#3 public opinion*”, “*proposed future*”, “*consumer acceptance*”, “*in vitro meat*” first appeared in 2000. Later, “*Anthropocene*”, “*construction*”, and “*clean meat*” appeared in 2018. Finally, “*nanotechnology*” emerged in 2020. Public perception of cell-cultured meat primarily centers on consumer research [25,27]. These breakthroughs are intricately tied to the technological aspects of cell-cultured meat, such as its transgenic nature, serum-free methods, and the integration of nanotechnology [68]. Consequently, public opinion surrounding cell-cultured meat is primarily concerned with the potential challenges and ethical considerations associated with this novel meat product [62,69]. Scholars have recently undertaken comparative studies on consumer acceptance across different countries, including China, the USA, and Italy [62,70]. Additionally, researchers have explored consumer preferences regarding plant-based and cultured meat-based burgers [40,71]. Furthermore, scholars have delved into the challenges and prospects associated with consumer acceptance of cell-cultured meat [72,73,74].

“*#4 animal welfare*” “*consumer perception*” appeared in 2017; “*innovation*”, “*pluripotent stem cell*”, and “*animal welfare*” appeared in 2018; “*quality*” and “*pork*” appeared in 2019; and “*nutritional composition*”, “*willingness to pay*”, and “*product*” appeared in 2020. Finally, “*3D printing*” appeared in 2022. This chronological progression suggests that animal welfare research has primarily centered on consumer intentions. Initially, the emphasis was on the welfare of animals in the context of cell-cultured meat research. However, as time passed, the focus gradually shifted towards improving the quality of cell-cultured meat and enhancing consumers’ willingness to pay. Recent research endeavors have also delved into enhancing technical support, such as the application of 3D printing, as a means of technological advancement [75].

“*#5 food safety*”, “*high moisture extrusion*”, “*safety*”, and “*food safety*” were the latest appearances in 2021, indicating a recent surge in scholarly interest in this domain. High-moisture extrusion plays a pivotal role in supporting the structural integrity of artificial meat [76] and facilitating the scalable production of cell-cultured meat to cater to various consumer demands. As cell-cultured meat advances, it explores food safety and emphasizes meeting consumer preferences [77]. This development should focus on technological advancements, assessing environmental impacts, and establishing a requisite regulatory framework for future large-scale production in food safety [78].

### 3.7. Keyword Emergent Analysis of Cell-Cultured Meat Research

**Figure 11:**
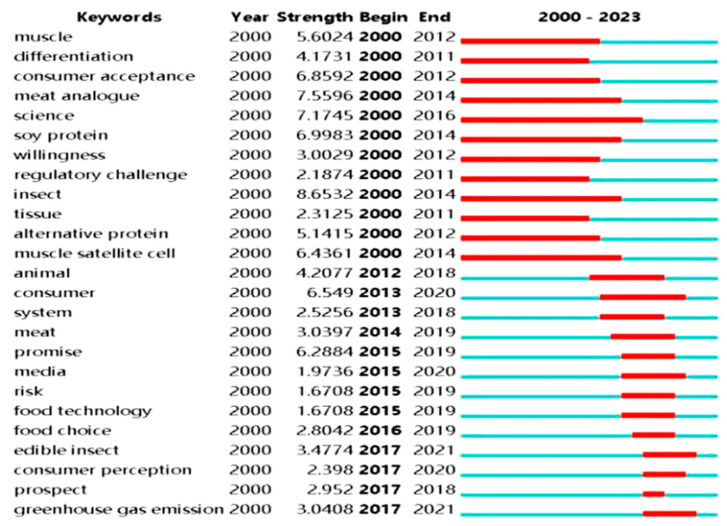
Top 25 emergent keywords for cell-cultured meat.

Emergent words appear frequently over a short period, reflecting the research focal points within a specific timeframe and aiding scholars in assessing the evolving trends in their field [55]. Citespace, a tool employed for this purpose, detects keywords within the field by tracking changes in word frequencies among evolving terms. Consequently, it discerns shifts in research hotspots and trends in the field. These alterations effectively mirror scholars’ research interests and highlight the field’s evolutionary trajectory concerning cell-cultured meat [55]. In this paper, we use Citespace software, configuring it with a top value 25. We designated “keywords” as the node type representing keywords. The column housing “*year*” denotes the respective year of the node, while strength quantifies the corresponding degree of emergence. The columns labeled ‘*beginning*’ and ‘*end*’ specify the onset and conclusion of the emergence, respectively. Employing these settings, we identified the top 25 emergent keywords. In Figure 11, the red line graphically illustrates each keyword’s prominence duration. The blue line represents the total duration of the selected time period.

We can delineate three distinct stages according to the evolution of keyword trends. The initial stage is from 2000 to 2013. During this phase, research primarily centers around biotechnology and animal-related aspects, focusing on technological perspectives. Researchers aim to comprehend the characteristics and applications of cell-cultured meat, exploring areas such as “muscle tissue”, “differentiation”, “consumer willingness”, “regulatory challenges”, “insects”, “alternative protein sources”, and “muscle satellite cells”. The second phase covers 2013 to 2019. The research landscape witnesses a shift. Key terms during this period include “*consumer*”, “*system*”, “*promise*”, “*media*”, “*risk*”, and “*food technology*”. This research phase delves into the natural environment and consumer attitudes [62,79], departing from purely technical concerns. The third and current phase, from 2019 to 2022, has honed its focus on broader issues, including “*food choices*”, “*edibility*”, “*insects*”, “*consumer perception*”, “*prospects*”, “*greenhouse gas emissions*”, and more. Research in this phase adopts a macroscopic approach, with a growing emphasis on environmental considerations such as food choices and greenhouse gas emissions [80]. We are in the third phase of the nascent stage of the cell-cultured meat industry. This phase primarily involves infusing social capital into the sector and attaining a particular scale in production and marketing [81]. Although some countries, such as Singapore and Israel, have companies selling cell-cultured meat products, the global industry remains at the pilot stage due to its relatively low production volume.

## 4. Discussion on Cell-Cultured Meat

This study analyzes 484 papers on cell-cultured meat available in the Web of Science database. We employ bibliometric visual analysis software to facilitate this analysis, enabling the creation of a comprehensive visual network analysis map. This map encompasses various aspects such as authorship, institutions, and countries involved in cell-cultured meat. Our primary objective is to shed light on the international cell-cultured meat research landscape from 2000 to 2022. Through this analysis, we seek to elucidate the fundamental contours of the field. Additionally, we delve into the subject terms and keywords associated with cell-cultured meat. This enables us to discern emerging research trends and areas of particular interest. The following is our further discussion based on the research literature.

### 4.1. Spatial Distribution and Naming Differences

**Figure 12:**
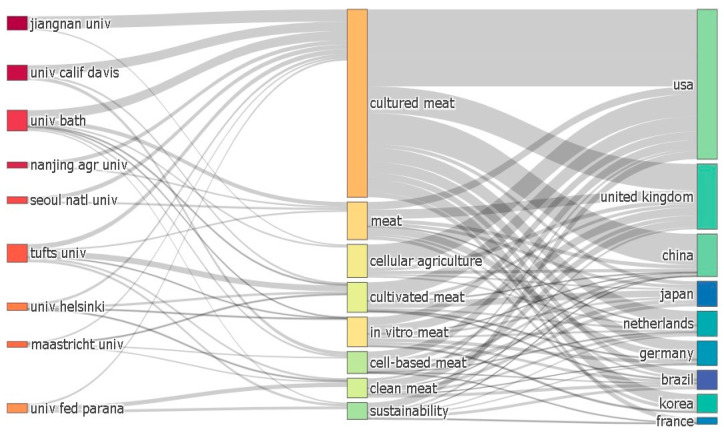
Institution, keywords, national Sangki diagram from 2000 to 2022.

(i) Regarding the spatial distribution of research authors in the field of cell-cultured meat, prominent locations include Ireland, Switzerland, the UK, Italy, The Netherlands, Belgium, and France in Europe; Singapore and China in Asia; Brazil in South America; and North America, encompassing the USA and Canada. Oceania, notably Australia, is also part of this landscape. The European region stands out in its early development and the high concentration of authors.
(ii) The Sankey diagram of issuing countries, keywords, and issuing organizations visualized by R language is shown in Figure 12 [51]. The results show that, firstly, the name “cultured meat” seems to be more prevalent in countries with many articles, such as the United States, the United Kingdom, and China. Secondly, the popularity of the names “cultivated meat”, “in-vitro meat”, “cell-based meat”, and “clean meat”, etc. range from the largest to the smallest, respectively. In addition, from an institutional perspective, in Asia, Jiangnan University and Nanjing Agricultural University in China prefer “cultured meat”, while Seoul National University from South Korea mainly uses “cultured meat”. In the Americas, the USA has used the term “cultured meat”. Most scholars from the University of California, Davis in the United States, and Parana Federal University in Brazil prefer “cultured meat”, while some scholars from the United States prefer to use the name “cultivated meat”. Some other American scholars prefer “cultured meat” and “cell-based meat”. However, Tufts University, which is also located in the United States, uses a broader range of nomenclatures and seems to prefer the name “cultivated meat” while also adopting the terms “cultured meat”, “in vitro meat, “and “clean meat”. The designation “in vitro meat” is less commonly used. However, institutions located in Europe, such as Helsinki University and Maastricht University, use all of the above nomenclature.

The difference in naming cell-cultured meat by institutions located in several continents of the world with a high volume of publications may be an exciting finding. We believe that the technical differences between different institutions are mainly related to the cell culture of the institution, but the consumer acceptance of different names may have different effects. This result may provide a reference and lesson to some extent for countries and institutions that have the intention of developing in the area of cell-cultured meat.

(iii) International Collaboration

There is extensive collaboration in cell-cultured meat involving the United States, the United Kingdom, China, and other nations. These collaborations span diverse topics.

First, America has partnered with countries like Belgium, the United Kingdom, South Africa, China, and Canada. Their collaborative efforts primarily revolve around consumer attitudes, acceptance, and perceived moral implications. For instance, joint studies with the United Kingdom and Belgium have explored the consumer sensory and nutritional aspects of cell-cultured meat [67]. They have also assessed consumer perceptions of cultured meat, using terms like “socially beneficial”, “high-tech”, and “identical to traditional meat” [26]. In collaboration with South Africa, American scholars have assessed consumer access to plant-based and cultured meat in South Africa. Collaboration with authors from China, Japan, and India in Asia has focused on evaluating consumer attitudes and perceptions, especially in regulated environments [82].

Second, British authors have collaborated with authors from various countries [56], including Canada and the United States in North America, China in Asia, Brazil in South America, and several European nations, such as The Netherlands, Italy, and France. Their collaborative research with North American authors has centered on consumer fears and perceptions’ impacts on cultured meat acceptance. Collaboration with Asian and South American authors has primarily explored consumer acceptance and the entrepreneurial ecosystem related to cell-cultured meat [64]. Collaboration with European authors has delved into cell-cultured meat’s history and technological advancements. For instance, Bryant conducted a comparative analysis of the cell-cultured meat market in France and Germany, highlighting its substantial market presence in European countries. They emphasized public concerns regarding antibiotics and food safety, bolstering artificial meat development.

Finally, Chinese authors have forged collaborations primarily with authors from the U.S., the UK, Korea, and India. These collaborations have been diverse, with partnerships with the U.S. and the UK focused on regulatory aspects, consumer acceptance, and identity labeling research. Collaborations with Asian counterparts, such as Korea and India, have centered on technological factors, such as integration into the biological processes of cultured meat. Additionally, several countries, including Belgium, Finland, Iran, Italy, and India, have initiated sporadic collaborations with authors from other nations in cell-cultured meat.

### 4.2. Regulatory Implications

Firstly, regulatory oversight for cell-cultured meat in the United States involves multiple agencies. The U.S. Food and Drug Administration (FDA) conducts inspections across various stages, including cell harvesting, processing, and labeling. Simultaneously, the U.S. Department of Agriculture (USDA) supervises cell collection, bank development, and maintenance, ensures biological inputs’ safety, and monitors acceptable production practices [83,84]. Additionally, the Food Safety and Inspection Service (FSIS) regulates post-harvest production and labeling aspects of cell harvesting [85].

Secondly, in Europe, cell-cultured meat is not classified as a subsidiary food product category within the European Union (E.U.) regulations. Instead, it is authorized as a novel food product subject to distinct laws and regulations. Notably, the UK holds a significant role in regulating cell-cultured meat. However, a key distinction lies in the final approval process, as the UK’s decision rests with a government ministerial official rather than the E.U.

In Asia, the Japan Association of Cellular Agriculture (JACA) actively advocates for changes in government agencies’ management styles and tools, offering recommendations to regulatory authorities to shape the food market’s oversight. China is in the process of proposing a regulatory framework for cell-cultured meat, primarily relying on the Food Safety Law, the Measures for the Administration of the Safety Review of New Food Ingredients, and the 2020 Health and Welfare Commission’s response to proposals related to cell-cultured meat within the National People’s Congress.

## 5. Conclusion and Research Outlook

Based on the cluster analysis of keywords and subject terms, we can summarize the research directions of scholars in this field from the clustering results. International research on cell-cultured meat holds promising prospects, characterized by the following key areas:

### 5.1. Conclusions

(i) Regarding publication time and volume changes, international research on cell-cultured meat from 2000 to 2022 can be categorized into two distinct phases: an initial period of fluctuating growth (2000–2013) and a subsequent phase of rapid expansion (2013–2022). Notably, the pivotal moment marking the transition occurred when the first cell-cultured meat burger was publicly sampled on a televised program in 2013. This event was a unique amalgamation of scientific communication, experimentation, and culinary showcase [66]. Since that groundbreaking moment in 2013, the discourse surrounding cell-cultured meat has witnessed a remarkable upswing. The overall trajectory of research and publications has been consistently upward, with a significant surge observed post-2013.
(ii) Several networks have emerged among research institutions dedicated to studying cell-cultured meat. Bath University has been the most prolific publisher in this field, followed by Maastricht University, Tufts University, Jiangnan University, ASTAR, University of Oxford, Nanjing Agricultural University, Seoul National University, Mosa Meat B.V., and the Swiss Federal Institute of Technology. Maastricht University exhibited the highest centrality. In descending order, the top 10 countries contributing to the cell-cultured meat literature are the USA, the UK, China, New Zealand, Germany, Korea, Australia, Canada, France, and Italy.
(iii) At the author level, three primary author groups have emerged in cell-cultured meat research. Group 1 includes notable authors such as Post and Lee. Authors like Park et al. represent Group 2, and Group 3 includes authors like Kaplan et al. Authors from the USA, the UK, China, and others have pivotal roles in advancing international cell-cultured meat research. Kaplan, Post, and Lee are central figures in this collaborative effort. However, there is ample room for enhancing international collaboration among authors from diverse countries in cell-cultured meat.
(iv) Regarding highly cited authors and journals in the Web of Science core collection, articles from the “Trends in Food Science & Technology” journal have received the most citations since 2000, accumulating 595 citations. The journal boasts an impact factor of 16.002 in 2021 and 17.835 over the past five years. Regarding research keywords, international cell-cultured meat studies primarily revolve around biomaterials, farming practices, land utilization, public opinion, animal welfare, food safety, and more.
(v) We reveal differences in the field of cell-cultured meat from the point of view of spatial distribution. First, the countries where there is research on cell-cultured meat are mainly located in Europe, the Americas, Asia and Oceania. Second, there are significant differences in terms of the scope and content of collaborations between the United States, England, and China. In addition, there are differences in regulatory policies in the Americas, Europe and Asia. In the Americas, the regulation of cell-cultured meat in the United States involves multiple agencies. In Europe, cell-cultured meat is authorized by the European Union as a novel food product and it is subject to different laws and regulations. In Asia, the Japanese Association for Cellular Agriculture (JACA) has an important regulatory role. In addition, China is proposing a regulatory framework for cell-cultured meat. Finally, this study reveals regional and institutional differences in nomenclature. “Cultured meat” is more popular among countries than other terms. Institutions in Asia use “cultured meat” more frequently; however, institutions in the Americas adopt “cultivated meat” and rarely adopt “in vitro meat”, and institutions in the European region have no particularly prominent tendency for nomenclature.

### 5.2. Potential Limitations

This paper describes a comprehensive situation in the development of cell-cultured meat over the past 20 years in both temporal and spatial dimensions. Meanwhile, it also provides a theoretical basis for the promotion and application of alternative proteins. However, there are still limitations in terms of the validity of the scope and the content of this study.

(i) The current study attempt to use the WOS core collection as a data source. Therefore, there may be a need to use other data sources as sources of analysis. As a result, we may have missed some relevant studies on cell-cultured meat. However, the WOS core collection, as a database with high international influence worldwide, has a large user base. Therefore, the currently selected databases can better reflect the overall situation of cell-cultured meat research. In addition, we set the search period from 2000–2022, so some literature before 2000 and after 2022 may be missed. However, we tried to cover as much of the literature as possible from the time we started this study, so the omissions are not enough to have a critical impact on the core results. At the same time, we used nine search terms closely related to cell-cultured meat to cover as much as possible all studies.
(ii) In terms of the content of the studies, we find that in recent years authors have been more detailed and specific about cell-cultured meat. The present study has elaborated to the best of its ability the temporal and spatial analysis of the progress of each research topic in the analysis of results section. Furthermore, some interesting results are drawn. Therefore, the temporal and spatial analysis of each research theme could be further refined in the future. For example, we can refine the elements of marketability related to the concerns about cell-cultured meat. So, further research could include a comparison of differences in willingness to pay for cell-cultured meat between different countries. Moreover, the variability of cell-cultured meat technology and regulation across countries is also a good direction, such as the mechanisms behind the differentiation in nomenclature.

### 5.3. Research Prospects

#### 5.3.1. The Research Content of Cell-Cultured Meat Is Expanding to the Multidisciplinary Cross-Cutting Fields

First, current research on cell-cultured meat focuses on consumption, technology, and the environment. In the future, with the large-scale development of cell-cultured meat, cell-cultured meat should be expanded to industry, economy, management, etc. Secondly, multidisciplinary research is conducive to consensus on using the term cell-cultured meat among different countries, organizations, and authors. There is still no uniform standardization and authoritative definition for the term, so the authoritative use of cell-cultured meat will help augment the discipline’s development prospects. Finally, the discipline should be expanded to emerging application scenarios, leveraging artificial intelligence software such as machine learning and neural networks and the ability to predict the possible environmental and social benefits and ecosystem function transformation of cell-cultured meat.

#### 5.3.2. The Cell-Cultured Meat Sector Should Strengthen the Overall Assessment of Market and Industry Development

Firstly, regarding the overall environmental value of cell-cultured meat, its impact on human environmental aspects currently needs to be clarified, including the contribution of the cell-cultured meat life cycle to global sustainability, because while cell-cultured meat may require fewer labor costs, it requires the depletion of more industrial sources. In addition, its ability to reduce greenhouse gas emissions is controversial. It also strengthens the socialization space required to produce and sell cell-cultured meat and the ecological impacts on society, such as the structure of traditional animal husbandry. Secondly, from the perspective of the scale of the industrial development of cell-cultured meat, despite some companies selling cell-cultured meat products in some countries, such as Singapore and Israel, the global industry remains at the pilot stage because of the relatively low production volume. Finally, based on the consumer perspective, more and more scholars have realized that cell-cultured meat may have ethical and psychological problems. In the future, the micro level should pay attention to whether the use of the media has a significant role in guiding the public’s views about cell-cultured meat.

#### 5.3.3. Constructing China’s Cell-Cultured Meat Technology and Regulatory System

First, according to the study results, there is still much room for China to intensify its international cooperation in terms of research on cell-cultured meat. Therefore, China should strengthen its cooperation with other countries further, increase investment in basic R&D, and build China’s core technology system for cell-cultured meat. Secondly, from the perspective of sustainable development of the cell-cultured meat industry, although cell-cultured meat has received wide attention in recent years, there is still no uniform specification on the naming and labeling of cell-cultured meat, so China should establish a standard system for cell-cultured meat as soon as possible. Finally, regarding regulatory mechanisms, reference should be made to developed countries’ regulatory mechanisms to improve the regulation of the cell-cultured meat industry. China should establish its own regulatory mechanism because of the advantages and shortcomings of developed countries’ regulatory mechanisms. From the government’s point of view, it should clearly define the regulatory departments, delineate the corresponding regulatory responsibilities, and clearly define the final approval decision to avoid regulatory overlap.

## Figures and Tables

**Table 1:** Top 10 co-cited authors in the literature.

Cited Times	Centrality	Cited Author-Name
452	0.07	Post, M.J. [9,20]
383	0.11	Stephens, N. [11]
322	0.10	Tuomisto, H.L. [15]
310	0.07	Bryant, C.J. [25]
295	0.05	[Anonymous]
268	0.13	Verbeke, W. [56]
266	0.11	Bhat, Z.F. [57]
248	0.06	Siegrist, M. [27]
231	0.05	Datar, I. [16]
206	0.09	Mattick, C.S. [21]

**Table 2:** Top 10 journals cited in the literature on high-frequency keywords.

Cited Times	Centrality	The Impact of Factors	Co-Cited Journal Name
595	0.04	2021 IF = 16.002; 5-year IF = 17.835	Trends in Food Science & Technology
549	0.04	2021 IF = 7.077; 5-year IF = 6.632	Meat Science
480	0.10	2021 IF = 3.752; 5-year IF = 4.069	PLoS ONE
440	0.02	2021 IF = 5.016; 5-year IF = 5.653	Appetite
384	0.04	2021 IF = 5.005; 5-year IF = 5.49	Frontiers in Sustainable Food Systems
322	0.09	2021 IF = 11.357; 5-year IF = 12.154	Environmental Science & Technology
307	0.05	2021 IF = 4.384; 5-year IF = 4.021	Journal of Integrative Agriculture
305	0.01	2021 IF = 5.561; 5-year IF = 5.94	Foods
300	0.08	2021 IF = 63.714; 5-year IF = 59.924	Science
299	0.02	2021 IF = 6.59; 5-year IF = 6.873	Frontiers in Nutrition

## Data Availability

The original contributions presented in the study are included in the article, further inquiries can be directed to the corresponding author.

## References

[1] FAO World Livestock 2011: Livestock in Food Security. https://www.fao.org/4/i2373e/i2373e00.htm.

[2] Sexton A.E., Garnett T., Lorimer J. (2019). Framing the future of food: The contested promises of alternative proteins. Environ. Plan. E Nat. Space.

[3] Godfray H.C.J., Aveyard P., Garnett T., Hall J.W., Key T.J., Lorimer J., Pierrehumbert R.T., Scarborough P., Springmann M., Jebb S.A. (2018). Meat consumption, health, and the environment. Science.

[4] Tuorila H., Hartmann C. (2020). Consumer responses to novel and unfamiliar foods. Curr. Opin. Food Sci..

[5] Henchion M., Hayes M., Mullen A., Fenelon M., Tiwari B. (2017). Future Protein Supply and Demand: Strategies and Factors Influencing a Sustainable Equilibrium. Foods.

[6] Kappenthuler S., Seeger S. (2019). Addressing Global Environmental Megatrends by Decoupling the Causal Chain through Floating Infrastructure. Futures.

[7] Onwezen M.C., Bouwman E.P., Reinder M.J., Dagevos H. (2021). A systematic review on consumer acceptance of alternative proteins: Pulses, algae, insects, plant-based meat alternatives, and cultured meat. Appetite.

[8] Ismail I., Hwang Y.H., Joo S.T. (2020). Meat analog as future food: A review. J. Anim. Sci. Technol..

[9] Post M.J., Levenberg S., Kaplan D.L., Genovese N., Fu J., Bryant C.J., Negowetti N., Verzijden K., Moutsatsou P. (2020). Scientific, sustainability and regulatory challenges of cultured meat. Nat. Food.

[10] Tuomisto H.L. (2019). The eco-friendly burger: Could cultured meat improve the environmental sustainability of meat products?. EMBO Rep..

[11] Stephens N., Di Silvio L., Dunsford I., Ellis M., Glencross A., Sexton A. (2018). Bringing cultured meat to market: Technical, socio-political, and regulatory challenges in cellular agriculture. Trends Food Sci. Technol..

[12] Bekhit E.D.A., Hopkins D.L., Fahri F.T., Ponnampalam E.N. (2013). Oxidative Processes in Muscle Systems and Fresh Meat: Sources, Markers, and Remedies. Compr. Rev. Food Sci. Food Saf..

[13] Kadim I.T., Mahgoub O., Baqir S., Faye B., Purchas R. (2015). Cultured meat from muscle stem cells: A review of challenges and prospects. J. Integr. Agric..

[14] Bonny S.P.F., Gardner G.E., Pethick D.W., Hocquette J.F. (2015). What is artificial meat, and what does it mean for the future of the meat industry?. J. Integr. Agric..

[15] Tuomisto H.L., Teixeira de Mattos M.J. (2011). Environmental Impacts of Cultured Meat Production. Environ. Sci. Technol..

[16] Datar I., Betti M. (2010). Possibilities for an in vitro meat production system. Innov. Food Sci. Emerg. Technol..

[17] Siegrist M., Sütterlin B. (2017). Importance of perceived naturalness for acceptance of food additives and cultured meat. Appetite.

[18] Mancini M.C., Antonioli F. (2019). Exploring consumers’ attitude towards cultured meat in Italy. Meat Sci..

[19] Hartmann C., Siegrist M. (2017). Consumer perception and behaviour regarding sustainable protein consumption: A systematic review. Trends Food Sci. Technol..

[20] Post M.J. (2012). Cultured meat from stem cells: Challenges and prospects. Meat Sci..

[21] Mattick C.S., Landis A.E., Allenby B.R., Genovese N.J. (2015). Anticipatory Life Cycle Analysis of In Vitro Biomass Cultivation for Cultured Meat Production in the United States. Environ. Sci. Technol..

[22] Hopkins P.D., Dacey A. (2008). Vegetarian Meat: Could Technology Save Animals and Satisfy Meat Eaters?. J. Agric. Environ. Ethics.

[23] Pluhar E.B. (2009). Meat and Morality: Alternatives to Factory Farming. J. Agric. Environ. Ethics.

[24] Benny A., Pandi K., Upadhyay R. (2022). Techniques, challenges and future prospects for cell-based meat. Food Sci. Biotechnol..

[25] Bryant C., Dillard C. (2019). The Impact of Framing on Acceptance of Cultured Meat. Front. Nutr..

[26] Pakseresht A., Kaliji S.A., Canavari M. (2022). Review of factors affecting consumer acceptance of cultured meat. Appetite.

[27] Siegrist M., Hartmann C. (2020). Consumer acceptance of novel food technologies. Nat. Food.

[28] Demartini E., Marescotti M.E., Amato M., Corradini A., Verneau F., Gaviglio A. (2024). Acceptance of alternative meats among different dietarian styles: An explorative analysis in Italy. Food Qual. Prefer..

[29] Stout A.J., Mirliani A.B., Rittenberg M.L., Shub M., White E.C., Yuen J.S., Kaplan D.L. (2022). Simple and effective serum-free medium for sustained expansion of bovine satellite cells for cell cultured meat. Commun. Biol..

[30] Dohmen R.G.J., Hubalek S., Melke J., Messmer T., Cantoni F., Mei A., Hueber R., Mitic R., Remmers D., Moutsatsou P. (2022). Muscle-derived fibro-adipogenic progenitor cells for production of cultured bovine adipose tissue. npj Sci. Food.

[31] Reis G.G., Heidemann M.S., Borini F.M., Maiolino Molento C.F. (2020). Livestock value chain in transition: Cultivated (cell-based) meat and the need for breakthrough capabilities. Technol. Soc..

[32] Califano G., Furno M., Caracciolo F. (2023). Beyond one-size-fits-all: Consumers react differently to packaging colors and names of cultured meat in Italy. Appetite.

[33] Loo E.J.V., Caputo V., Lusk J.L. (2020). Consumer preferences for farm-raised meat, lab-grown meat, and plant-based meat alternatives: Does information or brand matter?. Food Policy.

[34] Bryant C.J. (2020). Culture, meat, and cultured meat. J. Anim. Sci..

[35] Alexander P., Brown C., Arneth A., Dias C., Finnigan J., Moran D., Rounsevell M.D.A. (2017). Could consumption of insects, cultured meat or imitation meat reduce global agricultural land use?. Glob. Food Secur..

[36] Chriki S., Hocquette J.F. (2020). The myth of cultured meat: A review. Front. Nutr..

[37] Bryant C., Barnett J. (2018). Consumer acceptance of cultured meat: A systematic review. Meat Sci..

[38] Ben-Arye T., Levenberg S. (2019). Tissue engineering for clean meat production. Front. Sustain. Food Syst..

[39] Zhang G., Zhao X., Li X., Du G., Zhou J., Chen J. (2020). Challenges and possibilities for bio-manufacturing cultured meat. Trends Food Sci. Technol..

[40] Rubio N.R., Xiang N., Kaplan D.L. (2020). Plant-based and cell-based approaches to meat production. Nat. Commun..

[41] Van der Weele C., Feindt P., van der Goot A.J. (2019). Meat alternatives: An integrative comparison. Trends Food Sci. Technol..

[42] Santo R.E., Kim B.F., Goldman S.E., Dutkiewicz J., Biehl E.M.B., Bloem M.W., Neff R.A., Nachman K.E. (2020). Considering Plant-Based Meat Substitutes and Cell-Based Meats: A Public Health and Food Systems Perspective. Front. Sustain. Food Syst..

[43] Chriki S., Ellies O.M.P., Fournier D., Liu J., Hocquette J.F. (2020). Analysis of Scientific and Press Articles Related to Cultured Meat for a Better Understanding of Its Perception. Front. Psychol..

[44] Fernandes A.M., De Souza Teixeira O., Palma Revillion J.P., De Souza Â.R.L. (2020). Conceptual evolution and scientific approaches about synthetic meat. J. Food. Sci. Technol..

[45] Bryant C., Sanctorum H. (2021). Alternative proteins, evolving attitudes: Comparing consumer attitudes to plant-based and cultured meat in Belgium in two consecutive years. Appetite.

[46] Loveday S.M. (2019). Food proteins: Technological, nutritional, and sustainability attributes of traditional and emerging proteins. Annu. Rev. Food Sci. Technol..

[47] Lee H.J., Yong H.I., Kim M., Choi Y.-S., Jo C. (2020). Status of meat alternatives and their potential role in the future meat market—A review. Asian-Australas. J. Anim. Sci..

[48] Humpenöder F., Bodirsky B.L., Weindl I., Lotze-Campen H., Linder T., Popp A. (2022). Projected environmental benefits of replacing beef with microbial protein. Nature.

[49] Faust O., Hagiwara Y., Hong T.J., Lih O.S., Acharya U.R. (2018). Deep learning for healthcare applications based on physiological signals: A review. Comput. Methods Programs Biomed..

[50] Reiss J., Robertson S., Suzuki M. (2021). Cell Sources for Cultivated Meat: Applications and Considerations throughout the Production Workflow. Int. J. Mol. Sci..

[51] Chen Y., Lin M., Zhuang D. (2022). Wastewater treatment and emerging contaminants: Bibliometric analysis. Chemosphere.

[52] Van Eck N.J., Waltman L. (2010). Software survey: Vosviewer, a computer programfor bibliometric mapping. Scientometrics.

[53] Aria M., Cuccurullo C. (2017). Bibliometrix: An R-tool for comprehensive science mapping analysis. J. Informetr..

[54] Chen C. (2006). CiteSpace II: Detecting and visualizing emerging trends and transient patterns in scientific literature. J. Am. Soc. Inf. Sci. Technol..

[55] Freeman L.C. (1978). Centrality in social networks conceptual clarification. Soc. Netw..

[56] Verbeke W., Marcu A., Rutsaert P., Gaspar R., Seibt B., Fletcher D., Barnett J. (2015). ‘Would you eat cultured meat?’: Consumers’ reactions and attitude formation in Belgium, Portugal and the United Kingdom. Meat Sci..

[57] Bhat Z.F., Morton J.D., Mason S.L., Bekhit A.E.D.A. (2018). Role ofCalpain System in Meat Tenderness: A Review. Food Sci. Hum. Wellness.

[58] Guan X., Lei Q., Yan Q., Li X., Zhou J., Du G., Chen J. (2021). Trends and ideas in technology, regulation and public acceptance of cultured meat. Future Foods..

[59] Seah J.S.H., Singh S., Tan L.P., Choudhury D. (2021). Scaffolds for the manufacture of cultured meat. Crit. Rev. Biotechnol..

[60] Siegrist M., Sütterlin B., Hartmann C. (2018). Perceived naturalness and evoked disgust influence acceptance of cultured meat. Meat Sci..

[61] Siegrist M., Hartmann C. (2020). Perceived naturalness, disgust, trust and food neophobia as predictors of cultured meat acceptance in ten countries. Appetite.

[62] Bryant C., Barnett J. (2020). Consumer Acceptance of Cultured Meat: An Updated Review (2018–2020). Appl. Sci..

[63] Price D.J., Monaghan J.J. (2006). An energy-conserving formalism for adaptive gravitational force softening in sph and n-body codes. Mon. Not. R. Astron. Soc..

[64] Bryant C., Szejda K., Parekh N., Deshpande V., Tse B. (2019). A Survey of Consumer Perceptions of Plant-Based and Clean Meat in the USA, India, and China. Front. Sustain. Food Syst..

[65] Zhang J., Shi H., Sheng J. (2022). The effects of message framing on novel food introduction: Evidence from the artificial meat products in China. Food Policy.

[66] O’Riordan K., Fotopoulou A., Stephens N. (2016). The first bite: Imaginaries, promotional publics and the laboratory grown burger. Public Underst. Sci..

[67] Fraeye I., Kratka M., Vandenburgh H., Thorrez L. (2020). Sensorial and Nutritional Aspects of Cultured Meat in Comparison to Traditional Meat: Much to Be Inferred. Front. Nutr..

[68] Warner R.D. (2019). Review: Analysis of the process and drivers for cellular meat production. Animal.

[69] Dupont J., Fiebelkorn F. (2020). Attitudes and acceptance of young people toward the consumption of insects and cultured meat in Germany. Food Qual. Prefer..

[70] Palmieri N., Perito M.A., Lupi C. (2021). Consumer acceptance of cultured meat: Some hints from Italy. Br. Food J..

[71] Slade P. (2018). If you build it, will they eat it? Consumer preferences for plant-based and cultured meat burgers. Appetite.

[72] Verbeke W., Sans P., Van Loo E.J. (2015). Challenges and prospects for consumer acceptance of cultured meat. J. Integr. Agric..

[73] Szenderák J., Fróna D., Rákos M. (2022). Consumer acceptance of plant-based meat substitutes: A narrative review. Foods.

[74] Weinrich R., Strack M., Neugebauer F. (2020). Consumer acceptance of cultured meat in Germany. Meat Sci..

[75] Heidemann M.S., Taconeli C.A., Reis G.G., Parisi G., Molento C.F.M. (2020). Critical Perspective of Animal Production Specialists on Cell-Based Meat in Brazil: From Bottleneck to Best Scenarios. Animals.

[76] He J., Zhao Y., Jin X., Zhu X., Fang Y. (2021). Material Perspective on the Structural Design of Artificial Meat. Adv. Sustain. Syst..

[77] Fish K.D., Rubio N.R., Stout A.J., Yuen J.S., Kaplan D.L. (2020). Prospects and challenges for cell-cultured fat as a novel food ingredient. Trends Food Sci. Technol..

[78] Hadi J., Brightwell G. (2021). Safety of Alternative Proteins: Technological, Environmental and Regulatory Aspects of Cultured Meat, Plant-Based Meat, Insect Protein and Single-Cell Protein. Foods.

[79] Wilks M., Phillips C.J.C. (2017). Attitudes to in vitro meat: A survey of potential consumers in the United States. PLoS ONE.

[80] Lynch J., Pierrehumbert R. (2019). Climate Impacts of Cultured Meat and Beef Cattle. Front. Sustain. Food Syst..

[81] Messmer T., Klevernic I., Furquim C. (2022). A serum-free media formulation for cultured meat production supports bovine satellite cell differentiation in the absence of serum starvation. Nat. Food.

[82] Sheng J., Shi H., Zhang J. (2022). The role of environmental-related message on consumer acceptance of novel food production technology: An experimental investigation on artificial meat products. Environ. Sci. Pollut. Res..

[83] Servick K.U.S. (2018). Lawmakers float plan to regulate cultured meat. Science.

[84] Grossman M.R. (2019). United States: USDA and FDA formal agreement on regulation of cultured meat. Eur. Food Feed Law Rev..

[85] Sancar F. (2019). Agreement to Regulate Cell-Based Meat Products. JAMA.

